# Cardioprotective Effects of Oral Trimetazidine in Diabetic Patients With Anterior Wall Myocardial Infarction Treated with Thrombolysis

**DOI:** 10.14740/cr330w

**Published:** 2014-05-15

**Authors:** Mohamed Shehata

**Affiliations:** Department of Cardiology, Faculty of Medicine, Ain Shams University, Cairo, Egypt. Email: smarttmann@hotmail.com

**Keywords:** Trimetazidine, Thrombolysis, Diabetes mellitus, Cardiac enzymes

## Abstract

**Background:**

Trimetazidine is an anti-ischemic agent with anti-oxidant activity. This study sought to evaluate the impact of oral trimetazidine on extent of myocardial damage in diabetic patients who were presented with anterior wall ST-segment elevation myocardial infarction (STEMI).

**Methods:**

One hundred patients were prospectively enrolled, and then randomly assigned to receive oral trimetazidine (70 mg then 35mg bid) (group A, 50 patients) or placebo (group B, 50 patients), starting before thrombolysis. Serum creatine kinase-T and MB (CK-T and CK-MB) were measured serially. Degree of ST-segment resolution was recorded after 90 minutes. Left ventricle ejection fraction (LVEF) was assessed at baseline and after 6 months. Adverse events were recorded after thrombolysis and 6 months later.

**Results:**

Mean age of the study cohort was 59.05 ± 3.8 years (males: 60%). After 24 hours, 45 (90%) patients in group A vs. 10 (20%) patients in group B showed peaking of CK-T and CK-MB levels (P < 0.05). Both biomarkers’ levels were significantly higher in the placebo group at different sampling times. Complete resolution of ST-segment elevation was recorded in 35 (70%) patients in group A vs. 18 (36%) patients in group B (P < 0.05). Six months later, group A showed higher LVEF and fewer cardiac adverse events (P < 0.05).

**Conclusion:**

In diabetic patients receiving thrombolytic therapy for anterior wall STEMI, oral trimetazidine dosing was associated with less myocardial damage, earlier successful reperfusion, improvement of LVEF and less cardiac adverse events.

## Introduction

Trimetazidine (1-(2, 3, 4-trimethoxybenzyl) piperazine) is a cellular anti-ischemic agent that selectively inhibits the activity of the final enzyme of the fatty acid oxidation pathway, 3-ketoacylcoenzyme A thiolase. Administration of this drug leads to a switch in preference of the energy substrate, resulting in partial inhibition of fatty acid oxidation and increased glucose oxidation. Clinical studies had shown that trimetazidine has cardio-protective effects in the setting of myocardial ischemia including acute myocardial infarction (MI) [1-6]. Trimetazidine has been described as a cellular anti-ischemic agent [7]. Thrombolytic agents are highly effective in re-opening occluded coronary arteries and it is the accepted mechanism by which the myocardium is preserved and thus lives saved [8]. However, within few hours of the administration of a thrombolytic agent or placebo, there are more deaths in the active group than in the control. This is attributed to reperfusion injury, as suggested by experiments using animal models. The prognosis of patients with acute MI receiving thrombolytic therapy may be improved by treatment that could reduce the production or increase the elimination rate of free radicals [5]. It was demonstrated that trimetazidine prevents the deleterious effects of ischemia, reperfusion at both the cellular and the mitochondrial levels, and exerts potent antioxidant activity on various tissue preparations [9, 10]. Trimetazidine inhibits the excessive release of oxygen-free radicals, increases glucose metabolism, limits intracellular acidosis, protects adenosine triphosphate (ATP) stores, reduces membrane lipid peroxidation and inhibits neutrophil infiltration after ischemia-reperfusion [11, 12].

This study sought to evaluate the impact of trimetazidine oral loading on extent of myocardial damage (cardiac enzymes release) and success of reperfusion of the infarct-related artery (ST-segment resolution), in diabetic patients who were presented with anterior wall ST-segment elevation myocardial infarction (STEMI). Also, the impact of continued oral therapy on left ventricle (LV) systolic function, morbidity and mortality was evaluated after a 6-month follow-up period.

## Methods

### Study design and data collection

One hundred consecutive diabetic patients, carrying the diagnosis of anterior wall STEMI, were prospectively enrolled in this study. They were referred to the coronary care unit (CCU) in the period between January 2011 and July 2013. All patients received thrombolytic therapy within 6 h of onset of chest pain. Exclusion criteria included: prior history of acute coronary syndrome, cardiogenic shock, prior history of percutaneous coronary intervention (PCI) or coronary artery bypass graft surgery, congenital heart disease or any myocardial disease apart from ischemia, skeletal muscle disorders, limited life expectancy due to coexistent disease, for example malignancy, previous treatment with trimetazidine or presence of contraindications to oral trimetazidine use (for example, Parkinson’s disease and other motion disorders) [13] or thrombolytic therapy (for example, previous intracranial hemorrhage or recent major trauma or surgery) [14]. After enrollment and before thrombolysis, patients were randomly assigned in 1:1 fashion to either trimetazidine group (group A) or placebo group (group B) according to a computer-generated random series of numbers. Randomization was performed by block randomization (blocks of 10 patients). Group A patients received oral trimetazidine loading dose (70 mg) upon CCU admission (15 - 30 min before receiving thrombolytic therapy), followed by daily dosing (35 mg twice daily), continued for 6 months. Group B received a placebo formula for the same period of time. Physicians and medical staff in CCU were unaware of block randomization. All included patients were subjected to detailed history taking including drug-intake, baseline followed by serial 12-lead electrocardiogram (ECG) and transthoracic echocardiogram (TTE). All patients received fibrin-specific thrombolytic (fibrinolytic) therapy in the form of intravenous (IV) alteplase (tPA), administered as follows: a bolus of 15 mg, followed by 0.75 mg/kg over 30 min (up to 50 mg) and then 0.5 mg/kg over 60 min (up to 35 mg) [14]. Before inclusion, informed written consent was obtained from each patient and the study protocol was reviewed and approved by our local institutional human research committee, as it conforms to the ethical guidelines of the 1975 Declaration of Helsinki, as revised in 2008.

### Definition of risk factors of coronary artery disease

The presence of hypertension was defined as systolic blood pressure ≥ 140 mm Hg and/or diastolic blood pressure ≥ 90 mm Hg, previously recorded by repeated non-invasive office measurements, which led to life-style modification and/or intake of antihypertensive drug therapy [15]. Dyslipidemia was defined as LDL cholesterol > 100 mg/dL, and/or serum triglycerides > 150 mg/dL, and/or HDL cholesterol < 40 mg/dL (<50 mg/dL in women) [16].

### Baseline echocardiographic assessment

Assessment of regional and global LV systolic functions was performed in all patients by TTE using a General Electric Vivid 7 cardiac ultrasound machine (General Electric, Horten, Norway), equipped with harmonic imaging capabilities. A 2.5-MHz phased array probe was used to obtain standard 2D, M-mode and Doppler images. Patients were examined in the left lateral recumbent position using standard parasternal and apical views. Left ventricle ejection fraction (LVEF) by modified Simpson’s method, LV end diastolic volume (LVEDV), LV end systolic volume (LVESV) using bi-plane algorithm, wall motion abnormalities and wall motion score index (WMSI) were recorded for every patient, 24 - 48 h after thrombolysis and 6 months later. Regional wall motion was assessed according to the standard 17-segment model as recommended by the American Society of Echocardiography [17]. WMSI was calculated by dividing the sum of scores of the whole LV segments divided by the number of scored segments. Images were digitized in cine-loop format, and saved for subsequent playback and analysis. Views were analyzed by a single echocardiographer employing the software program of the echocardiography machine and blinded to the study protocol. Regional wall motion abnormalities were visually assessed for each segment individually, considering both endocardial excursion and systolic thickening. Each segment was graded according to the semi-quantitative scoring system described by Cerqueira et al [17]. Segments with poorly defined endocardial borders for 50% or more of their length were considered non-visualized and assigned a score of 0 [18]. Wall thickening was assessed at a distance of at least 1 cm from the adjacent segment, to minimize the effect of tethering [19]. Images were digitized in cine-loop format, and saved for subsequent playback and analysis. Views were analyzed by a single echocardiographer (for both baseline and follow-up assessments) employing the software program of the echocardiography machine and was blinded to the study protocol.

### Medical treatment and laboratory work-up

Blood sampling for creatine kinase-total (CK-T) and creatine kinase-MB (CK-MB) isoenzyme levels was done just before thrombolysis and then repeated after 12 h followed by daily sampling till normalization of serum levels of both markers. Measurements were performed with the use of a chemiluminescence immunoassay and Elecsys 2010 analyzer from Roche Diagnostics Laboratory. The upper limit of normal reference range was 220 U/L for CK-T and 8.8 U/L for CK-MB.

All patients received regular anti-ischemic medical treatment including: aspirin, clopidogrel, beta-blockers, angiotensin converting enzyme inhibitors and nitrates. All patients received enoxaparin upon admission (30 mg IV bolus followed by 1 mg/kg/12 h, given subcutaneously) [14]. They were followed up closely for recording of post-MI complications (pump failure, arrhythmias, mechanical complications, LV thrombus, and so on), if any.

### Twelve-lead ECG monitoring

A 12-lead ECG was recorded upon presentation to CCU (baseline ECG), 90 min after thrombolysis and at 12 h intervals throughout the CCU stay period. Additionally, three-lead ECG monitoring was constantly followed up especially for occurrence of malignant arrhythmias. ST-segment resolution was judged using the ECG done 90 min after thrombolysis. The sum of ST-segment elevation 20 ms after the J point was calculated and compared with the baseline ECG. The percent resolution was categorized as complete (≥ 70%), partial (30% to < 70%), or none (< 30%) [20].

### Recording of clinical adverse events

In-hospital adverse events were recorded for both study groups including: in-hospital mortality, heart failure, malignant ventricular arrhythmias, ventricular septal rupture, occurrence of LV thrombus, recurrence of angina pain, re-infarction and cerebrovascular stroke. Six months’ data collection included recording of cardiac mortality, hospitalization due to heart failure and re-infarction.

### Statistics

All continuous variables were statistically described in terms of mean ± standard deviation (SD). Categorical variables were described with absolute and relative (percentage) frequencies. Comparison of continuous variables between the study groups was done using Student’s *t*-test. For comparing categorical data, Pearson Chi-square and Fisher exact tests were performed. P values were used to describe significance. All statistical calculations were done using Statistical Package for Social Sciences (SPSS for Windows) software (version 15.0, SPSS Inc., Chicago, IL, USA).

## Results

### Baseline clinical characteristics

A total of 100 consecutive diabetic patients with anterior wall STEMI were prospectively enrolled in this study. All patients received IV alteplase, in addition to adjunctive anti-thrombotic therapy and anti-ischemic therapy. The current study comprises 50 patients randomly assigned to trimetazidine group (group A) receiving the drug according to the previously described dosing regimen, and 50 others randomly assigned to the placebo group (group B). The mean age of the whole study cohort was 59.05 ± 3.8 years, 60 (60%) being male patients. The two study groups were matched regarding age, gender, risk factors of coronary artery disease and baseline TTE data. No statistically significant difference was found between the two groups concerning mean baseline glycated hemoglobin (HbA1c) level. Table 1 shows baseline characteristics of the two study groups.

**Table 1:** Baseline Characteristics of the Two Study Groups

Variable	Group A (n = 50)	Group B (n = 50)	P value*
Age (years)	59.6 ± 5.4	58.5 ± 2.3	> 0.05
Males	31 (62)	29 (58)	> 0.05
Hypertension	24 (48)	25 (50)	> 0.05
Dyslipidemia	20 (40)	21 (42)	> 0.05
Smoking	15 (30)	17 (34)	> 0.05
Baseline CK-T (U/L)	222 ± 8.4	218 ± 6.4	> 0.05
Baseline CK-MB (U/L)	8.9 ± 2.4	9.3 ± 3.6	> 0.05
HbA1c (%)	7.2 ± 0.2	7.1 ± 0.3	> 0.05

CK-T: creatine kinase-total; CK-MB: creatine kinase-MB isoenzmye; HbA1c: glycated hemoglobin. Categorical variables are presented as number (percentage). Continuous variables are presented as mean ± standard deviation. *Pearson Chi-square and Student’s t-test.

### Cardiac enzymes and ST-segment resolution

**Figure 1:**
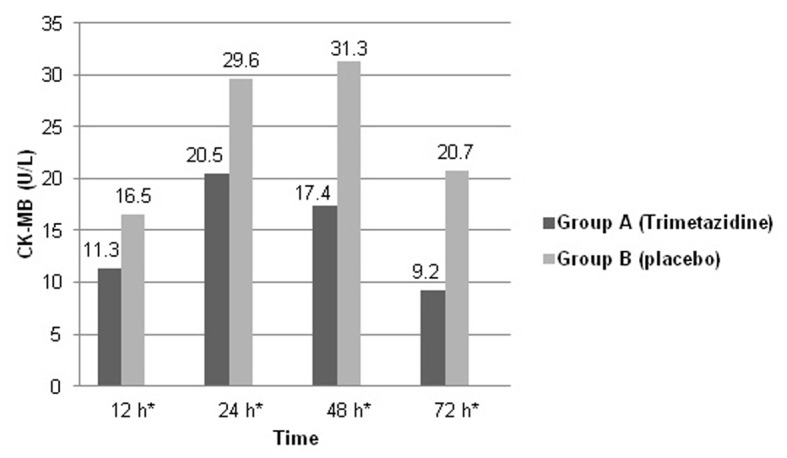
Graphic presentation showing changes in mean CK-MB levels in both study groups in the first 72 h after thrombolysis. *P < 0.05 (Student’s t-test).

**Figure 2:**
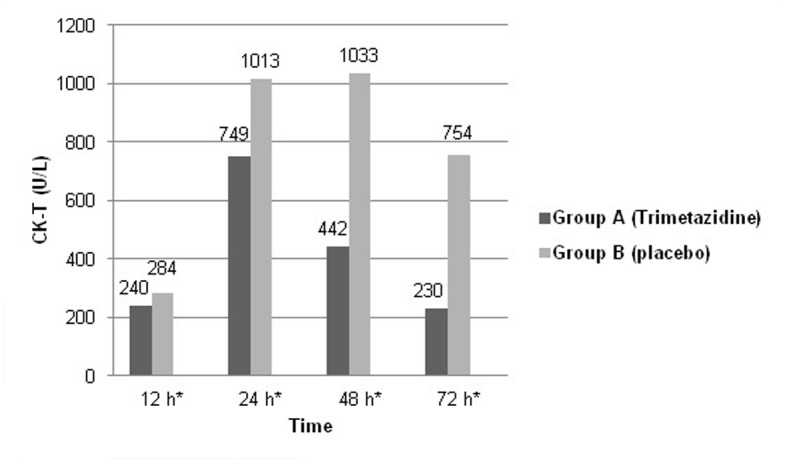
Graphic presentation showing changes in mean CK-T levels in both study groups in the first 72 h after thrombolysis. *P < 0.05 (Student’s t-test).

**Figure 3:**
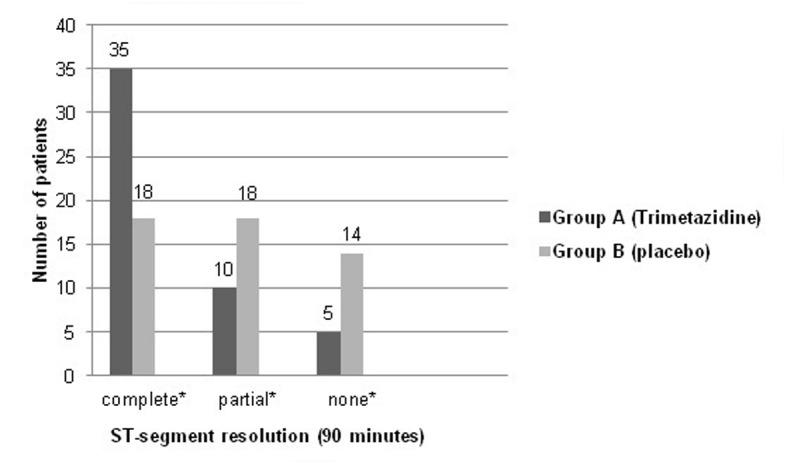
Graphic presentation showing ST-segment changes (90 min after thrombolysis) in both study groups. *P < 0.05 (Pearson Chi-square test).

Mean baseline CK-T and CK-MB levels were not significantly different between both study groups. After 24 h, 45 (90%) patients in group A versus 10 (20%) patients only in group B showed peaking of CK-T and CK-MB levels (P < 0.05). Also, both biomarkers’ levels were recorded to be significantly higher in the placebo group at different sampling times. Patients in group A showed almost normalized cardiac enzymes levels after 72 h (mean CK-MB: 9.2 ± 1.2; CK-T: 230 ± 6.5 U/L), while group B patients almost reached enzymes’ normalization after 96 h (mean CK-MB: 9.5 ± 2.3 U/L; CK-T: 228.3 ± 6.4 U/L). Mean CK-T and CK-MB levels recorded in the first 72 h after thrombolysis are shown in Figures 1 and 2. ECG done 90 min after thrombolysis revealed complete resolution of ST-segment elevation in 35 (70%) patients in group A, while this was recorded in 18 (36%) patients only in group B (P < 0.05). ST-segment changes in both study groups are shown in Figure 3.

### Echocardiographic data

Echocardiographic data obtained within 24 - 48 h after thrombolysis did not differ significantly between both study groups. However, after 6 months, group A showed a highly significant improvement in LVEF, compared to baseline data. Also, there was a significant reduction in LVESV and WMSI. Echocardiographic data collection was done for 47 patients only in group B (three cases of cardiac mortality were recorded during the follow-up period). Analysis of intra-observer variability revealed a close correlation between repeated recordings of regional wall motion by the single operator, with a correlation coefficient r of 0.91. Echocardiographic data are summarized in Table 2.

**Table 2:** Echocardiographic Data of Both Study Groups (24 - 48 h After Thrombolysis and 6 Months Later)

	Baseline	After 6 months	P value*
Group A (n = 50)			
LVEF (%)	33.1 ± 4.5	39.5 ± 5.9	< 0.001
LVEDV (mL)	183 ± 12.7	181.8 ± 13	> 0.05
LVESV (mL)	121.8 ± 9.2	110.2 ± 13	< 0.05
WMSI	1.64 ± 0.3	1.29 ± 0.2	< 0.05
Group B (n = 47)			
LVEF (%)	33.4 ± 3.5	33.6 ± 3.4	> 0.05
LVEDV (mL)	181 ± 19.9	180.7 ± 18	> 0.05
LVESV (mL)	113.7 ± 11.7	110 ± 22	> 0.05
WMSI	1.7 ± 0.3	1.64 ± 0.4	> 0.05

LVEF: left ventricle ejection fraction, LVEDV: left ventricle end diastolic volume; LVESV: left ventricle end systolic volume; WMSI: wall motion score index. All variables are presented as mean ± standard deviation. *Student’s t-test.

### Clinical adverse events (in-hospital and follow-up)

There were no recorded cases of mortality or mechanical complications during the in-hospital stay among patients in both study groups. There was no significant difference between both groups concerning incidence of adverse events during hospitalization. However, data recording after 6 months revealed higher incidence of cardiac mortality, mostly sudden cardiac death, hospitalization for heart failure and re-infarction (managed medically) in group B (P < 0.05). These data are summarized in Table 3. All patients were followed up regularly through cardiology out-patient clinic and phone calls with adjustments of medical treatment, whenever needed. Glycated hemoglobin was checked at the end of the follow-up period with no significant difference between both groups (group A: 6.9 ± 0.22%, group B: 7 ± 0.12, P > 0.05).

**Table 3:** Recorded Adverse Events for Both Study Groups (During In-Hospital Stay and After 6 Months)

Variable	Group A (n = 50)	Group B (n = 50)	P value*
In-hospital			
HF	5 (10)	7 (14)	> 0.05
Sustained VA	4 (8)	5 (10)	> 0.05
LV thrombus	4 (8)	6 (12)	> 0.05
Recurrence of angina	8 (16)	6 (12)	> 0.05
Re-infarction	0	2 (4)	> 0.05
Cerebrovascular stroke	0	1 (2)	> 0.05
Six months			
Cardiac mortality	0	3 (6)	< 0.05
Hospitalization for HF	3 (6)	10 (20)	< 0.05
Re-infarction	1 (2)	5 (10)	< 0.05

HF: heart failure; VA: ventricular arrhythmia; LV: left ventricle. All variables are presented as number (percentage). *Pearson Chi-square and Fisher exact tests.

## Discussion

The current study presented two points of strength concerning pre-treatment with oral trimetazidine in diabetic patients, receiving thrombolytic therapy as a reperfusion strategy for management of STEMI. The first point was shown when the group of patients pretreated with trimetazidine showed significantly less myocardial damage evidenced by earlier peaking and lower total amount of cardiac enzymes’ (CK-T and CK-MB) release during the hospitalization period. Furthermore, the same group of patients (continued trimetazidine therapy for 6 months) showed significant myocardial function recovery evidenced by significant improvement of LVEF after 6 months. Interestingly, that was associated with less incidence of cardiac mortality, hospitalization for heart failure and repeated MI. The daily dose of trimetazidine used in the current study (35 mg twice daily) was reviewed by European Medicines Agency in June 2012 and was reported to improve ischemic symptoms [13]. The results of the current study add evidence to the safety of this daily dose. However, the conventionally used dose is 20 mg thrice daily. The author sought to select patients with anterior wall STEMI for inclusion, in order to obtain more pronounced results especially concerning cardiac enzymes’ levels and LVEF change.

It is worth highlighting that both groups of patients showed a comparable baseline risk profile. Moreover, there was no significant difference recorded regarding baseline CK-T and CK-MB levels. Also, both groups seemed to have a comparable glycemic control evidenced by almost similar mean baseline HbA1c levels. The favorable effect of trimetazidine on myocardial necrosis could be explained by both its metabolic and biological effects. Trimetazidine had been shown to act as a cellular anti-ischemic agent without any hemodynamic effects [21]. It acts by improving cardiac energy metabolism by switching ATP production from lipid to glucose oxidation, thus enhancing intra-mitochondrial coupling and favoring a more efficient mode of ATP production per mole of oxygen. Moreover, trimetazidine reduces intracellular acidosis and protects against oxygen free radical induced toxicity. The drug therefore directly protects myocyte structure and function and increases cell resistance to hypoxic stress [11, 22, 23]. The potent antioxidant effect of trimetazidine had been demonstrated in minimizing myocardial, renal and hepatic ischemia-reperfusion injury [24-26]. Moreover, it was shown that pre-treatment with trimetazidine was effective in reducing the size of the infarct that develops in the blood-perfused rabbit model of myocardial ischemia [27]. Another animal experiment demonstrated that trimetazidine could limit lethal ischemia-reperfusion injury by inhibiting mitochondrial permeability transition pore opening, which represents a crucial event in cardiomyocyte death following myocardial ischemia-reperfusion [28].

The pattern of cardiac biomarkers release in both study groups declared that trimetazidine group of patients exhibited less myocardial damage and put the cardioprotective effect of this drug in a clear place to recognize. This appeared to be also related to earlier successful reperfusion of the infarcted myocardium. Evidence was added upon recording that the vast majority of trimetazidine group of patients showed complete ST-segment resolution 90 min after receiving the fibrinolytic therapy.

Although, data recording concerning in-hospital adverse events revealed no significant difference between both study groups, the question was: how about an extended follow-up period for myocardial systolic function and clinical adverse events? The answer after 6 months interestingly revealed a significant improvement of LV systolic function in trimetazidine group of patients, translating the acute cardioprotective effect of trimetazidine into a more pronounced form. Moreover, the same group of patients had a significantly less eventful follow-up period, with no recorded mortality.

### Comparison with other studies

The cardioprotective anti-ischemic effect of trimetazidine was previously demonstrated in animals [12, 26, 29] and human beings undergoing PCI [30-33]. Limited studies had evaluated the assumed cardioprotective effects of trimetazidine in patients receiving thrombolytic therapy for STEMI management. This might be due to the well established role of mechanical reperfusion strategy, in addition to wide availability of primary PCI facilities in many countries especially in the last decade. However, in many developing countries especially in rural areas, these facilities are not widely provided and patients’ transfer to primary PCI centers, if any, remains costy, time-consuming and sometimes not available. Thrombolytic therapy remains the only reperfusion aid available in these areas. Similar to the present study, a prior study by Di Pasquale et al had previously reported that oral trimetazidine administration shortly before receiving thrombolytic therapy led to earlier peaking and less release of cardiac biochemical markers. Moreover, their study results showed that continued drug administration was associated with less cardiac mortality and favorable LV remodeling process (decreased LVESV) [6]. Another study reported the favorable impact of oral trimetazidine use on ST-segment resolution and creatine kinase release in the same clinical setting [34]. However, these studies were not targeting diabetic patients and the used oral dose of trimetazidine (20 mg thrice daily) was different from that used in the current study. Furthermore, their study protocols did not include detailed recording of in-hospital or post-hospitalization adverse events. Similar to the present study, previous studies had reported improvement of LVEF in diabetic patients with ischemic LV systolic dysfunction after regular oral intake of trimetazidine [35-37]. Free radicals are thought to participate in reperfusion arrhythmias that could be detrimental to the prognosis of the patient [38]. A previous study showed a trend in favor of oral trimetazidine (60 mg on admission then 20 mg for 5 days) in reducing the extent of severe reperfusion arrhythmias [4]. The current study did not reach the same conclusion upon analysis of in-hospital data. However, three patients in the placebo treated group died during the follow-up period raising the possibility of occurrence of malignant ventricular arrhythmias in this group. In contrast to the current study, EMIP-FR study group showed that trimetazidine use in patients treated with thrombolysis was not associated with decreased cardiac mortality after a long-term follow-up period. Trimetazidine receivers also showed higher incidence of in-hospital ventricular arrhythmias, compared to the placebo group [5]. However, EMIP-FR study protocol was different from the one adopted in the current study in many points, for example IV trimetazidine infusion (48 h) was rather used with no oral doses given thereafter. The authors themselves stated that the regimen used in this study may be inappropriate in terms of dose, site of delivery and/or timing. Also, various types of thrombolytics were used (fibrin and non-fibrin specific), in addition to relatively small percentage (about 12% of each study arm) of diabetic patients were included. Moreover, assessment of echocardiographic parameters including LV systolic function was not included in the study protocol.

To the best of the author’s knowledge, no previous trials addressed the cardioprotective effects of oral trimetazidine in diabetic patients receiving alteplase for management of STEMI. The current study highlighted that less myocardial damage in the acute clinical setting, less eventful 6 months follow-up period and evident recovery of LV systolic function were associated with oral trimetazidine use.

### Clinical implications

Trimetazidine is a well-tolerated drug, widely available in the oral formula. The results of the current study could add benefit to diabetic patients suffering from acute STEMI, commonly encountered by cardiologists in emergency rooms. The author hypothesizes that oral loading with trimetazidine (70 mg) given shortly before thrombolysis in diabetic patients, could help minimize myocardial damage. Additionally, oral daily dosing (35 mg twice daily) of the drug could augment the initial cardioprotective effect resulting in improved LV systolic function and less adverse events thereafter.

### Limitations of the study

The results presented in this study only apply for patients defined by inclusion and exclusion criteria. Extended follow-up (more than 6 months) of myocardial systolic function and adverse events were not included in the current study protocol. The functional aspect of the cardioprotective effect of trimetazidine was not assessed in the current study. This was outside the scope of the study protocol. Large-scale studies are still required to evaluate the long-term functional impact, for example exercise tolerance, of oral trimetazidine administration in similar clinical settings.

### Conclusion

In diabetic patients receiving alteplase (tPA) for management of anterior wall STEMI, oral loading followed by regular daily trimetazidine dosing was associated with less myocardial damage and earlier successful reperfusion, as compared with placebo treatment. Moreover, a 6-month follow-up period showed significant improvement in LV systolic function and less cardiac mortality.
